# Catalysts Based on Strontium Titanate Doped with Ni/Co/Cu for Dry Reforming of Methane

**DOI:** 10.3390/ma14237227

**Published:** 2021-11-26

**Authors:** Adrian Mizera, Andrzej Kowalczyk, Lucjan Chmielarz, Ewa Drożdż

**Affiliations:** 1 Faculty of Materials Science and Ceramics, AGH University of Science and Technology, 30-059 Kraków, Poland; edrozdz@agh.edu.pl; 2 Faculty of Chemistry, Jagiellonian University, 31-007 Kraków, Poland; kowalczy@chemia.uj.edu.pl (A.K.); chmielar@chemia.uj.edu.pl (L.C.)

**Keywords:** dry reforming of methane, SrTiO_3_, nickel catalyst

## Abstract

Two series of strontium titanates doped with Ni, Co, or Cu with general formula of SrTi_1-x_Me_x_O_3_ for Sr-stoichiometric and Sr_0.95_Ti_1−x_Me_x_O_3_ for Sr-non-stoichiometric materials (where Me = Ni, Co or Cu and x were 0.02 and 0.06) were obtained by the wet chemical method. The samples were calcinated at 900, 950, and 1050 °C and characterized in terms of their structural properties (XRD), the possibility of undergoing the reduction and oxidation reactions (TPR/TPOx), and catalytic properties. All obtained materials were multiphase and although the XRD analysis does not confirm the presence of Ni, Co, and Cu oxides (with one exception for Cu-doped sample), the TPR/TPOx profiles show reduction peaks that can be attributed to the reduction of these oxides which may at first appear in an amorphous form. Catalytic tests in dry reforming of methane reaction showed that the highest catalytic activity was achieved for Ni-doped materials (up to 90% of CH_4_ conversion) while Co and Cu-doped samples showed only a very slight catalytic effect. Additionally, the decrease in methane conversion with an increasing calcination temperature was observed for Ni-doped strontium titanates.

## 1. Introduction

Mixed oxides with a perovskite structure are one of the most extensively studied groups in the field of inorganic materials science or chemistry. Perovskites-type oxides can be described using general formula of ABO_3_, where larger A-site cations have a 12-fold coordination and smaller B-site cations are six-fold coordinated. Additionally, perovskites can crystallise in several crystal systems, mainly cubic, tetragonal and orthorhombic. Strontium titanate (STO) is an example of a perovskite material with properties that can be easily modified by changing the chemical composition—doping. From an applicational point of view, one of the most attractive features is its mixed ionic-electronic conductivity (MIEC) which is caused by proper doping of the perovskite structure with donor or acceptor elements [1]. Such properties are desired in case of anode materials for SOFC technology, which has gained a lot of interest due to its relatively high efficiency and environmental advantages. Although the most commonly used anodic material in SOFC devices—Ni/YSZ cermet (YSZ—yttria stabilised zirconia)—meets many material requirements for SOFC anode material (e.g., mixed conductivity, high open porosity over 30 vol. % and catalytic activity towards hydrogen oxidation reaction), there are some problems including poor resistance for coking, sulphur poisoning and the tendency of Ni particles to agglomerate at operating temperatures (700–900 °C) [2,3,4]. Due to above-mentioned disadvantages of the Ni/YSZ cermet, it is necessary to use purified hydrogen to power the cell, which increases the fuel mixture costs. In addition to the classical approach, where pure hydrogen is used as a fuel, research is being conducted on the production of the hydrogen *in statu nascendi* using natural gas inside the cell and thus replacing the hydrogen fuel with a gas mixture containing, e.g., methane and carbon dioxide [5]. The dry reforming of methane (DRM) is an endothermic chemical process which allows for the conversion of methane and carbon dioxide into hydrogen and carbon monoxide according to the following equation: $CH_{4}+CO_{2}⇄2CO+2H_{2}$ (ΔH^O^_298K_ = 247 kJ/mol). The combination of SOFC technology and internal dry reforming of methane not only creates the possibility for the extension of the fuel range and cost reduction but also leads to the challenge of creating material that is (in addition to the requirements for SOFC mentioned) catalytically active in the DRM reaction. The most common materials used to catalyse the DRM reaction are the composites based on nickel, nickel alloys or noble metals, such as Rh or Pt (usually 5–15 wt.%) deposited on oxide support [6]. In the case of noble metals, which exhibit a good resistance for coking, price and abundance are an issue. For the cheaper nickel, the aforementioned low-coking resistance and agglomeration tendency are the major problems. Carbon deposition results from the following reactions catalysed also by nickel and taking place in parallel to the main DRM reaction:

- Boudouard reaction: $2CO⇄C_{\left(s\right)}+CO_{2}$
- Reduction of CO with H: $CO+H_{2}⇄C_{\left(s\right)}+H_{2}O$
- Decomposition of methane: $CH_{4}⇄C_{\left(s\right)}H_{2}C_{\left(s\right)}+2H_{2}$

The replacement of nickel by other non-noble elements creates an opportunity to obtain a relatively cheap and highly active catalyst for the DRM process. Recent attempts to obtain anodic material for biogas-fuelled SOFC devices involve the modification of common Ni/YSZ cermet by infiltration of gadolinium doped ceria [7] copper and cobalt co-doped ceria [8] or even iridium doped ceria [9]. Exploiting the potential of doped perovskites, due to their chemical stability and good electrochemical properties, is another direction in the search for alternative materials for SOFC anodes. In the literature, there are a few articles devoted to the study of such materials in terms of their use as anodes in SOFCs powered by biogas [10,11], although doped SrTiO_3_ materials have already been tested for application as an anodic material for hydrogen-fuelled SOFCs, including ytterbium [12], niobium [13,14], yttrium [15] and lanthanum-doped [16] strontium titanates.

In our work, we decided to examine the structural and catalytic properties of strontium titanates doped with 2 and 6 mol.% of Ni, Co or Cu. The aforementioned susceptibility of Ni to coking prompted us to use Co and Cu as admixtures of strontium titanate and to check and compare the catalytic activity of materials based on Ni, Co and Cu doped strontium titanate. An additional motivation was the literature reports on the increased (compared to Ni) resistance to coking of cobalt/copper, although it is worth noting that it does not have to translate directly into a higher catalytic activity of materials based on Co-doped SrTiO_3_. In our research, we also wanted to investigate the effect of low non-stoichiometry in the strontium subnetwork on the increase in catalytic activity through the postulated ex-solution process. Such a process is based on the reversible introduction and removal of elements from the perovskite structure during oxidation or reduction processes, respectively [17]. Additionally, the influence of different calcination temperatures (900, 950 and 1050 °C) on both phase composition and catalytic activity towards the DRM reaction was investigated.

## 2. Materials and Methods

• SYNTHESIS

Two series of nickel, cobalt and copper-doped strontium titanates with the general formula of SrTi_1−x_Me_x_O_3_ (Sr-stoichiometric; labeled as STMe) and Sr_0.95_Ti_1−x_Me_x_O_3_ (Sr-non-stoichiometric; labelled as SvTMe), where Me means Ni, Co or Cu and x = 0.02 and 0.06 (labelled: STMe2; STMe6; SvTMe2; SvTMe6), were prepared using the modified sol-gel method. The first stage of synthesis was the dissolution of citric acid monohydrate (min. 99.5%, Avantor) in anhydrous methanol at 50 °C. The total molar ratio of citric acid to the sum of cations (Sr, Ti and Ni, Co or Cu) was set to be 3:2. Next, titanium (IV) isopropoxide (98+%, Acros Organics, Waltham, MA, USA) was added to the mixture and stirred for 2 h until a clear straw-colored solution was obtained. Subsequently, the appropriate volume of Sr(NO_3_)_2_ solution was added, followed by the addition of Ni(NO_3_)_2_, Co(NO_3_)_2_ or Cu(NO_3_)_2_ (min. 99.8%, Avantor, Radnor, PA, USA) solution depending on the type of dopant used in a specific sample. The sol prepared by this method was heated on a hot plate at 120 °C to evaporate the solvent (c.a. 3 h) and then at 250 °C overnight to partially decompose the nitrates and remove the organic residue. After cooling to room temperature, the xerogel was ground in agate mortar and calcined at 900, 950 and 1050 °C (heating rate 1 deg∙min^−1^) for 3 h in the air flow.

• EXPERIMENTAL

XRD analyses were carried out by using a Panalytical X’Pert Pro diffractometer (Malvern Panalytical Ltd, Malvern, UK) equipped with a Cu-K_α_ radiation source over 20–90°. The Rietveld refinement analysis (High Score Plus program, version 3.0,5, Malvern Panalytical, Malvern, UK) was used to determine the share of particular phases in samples.

The microstructure of the samples was observed using scanning electron microscope Nova Nano SEM 200 FEI (Oxford Instruments, Abingdon, UK).

The specific surface area (BET) measurements were made on an ASAP 2010 device (Micromeritics, Norcross, GA, USA). The samples were degassed at 150 °C for 24 h before measurement.

Temperature-programmed reduction (TPR) and oxidation (TPOx) were carried out by using ChemiSorb 2750 apparatus (Micromeritics, Norcross, GA, US). The mass of the samples was similar in all cases (approx. 150 mg). All the samples were placed in quartz reactor in a flow of 5%H_2_/95%Ar or 5%O_2_/95%Ar (in both cases the flow rate was 40 mL/min). Each TPR measurement were preceded by degassing the sample at 200 °C for 20 min.

• CATALYTIC SET UP

The catalytic activity performance of the samples in the DRM reaction was studied in a fixed-bed flow quartz microreactor system with detection of the reaction mixture by a quadruple mass spectrometer (Prevac, Rogów, Poland) connected directly to the reactor outlet. The following gas mixtures were used during the catalytic test: 1% CO_2_ in He and 1% CH_4_ in He, total flow rate of 40 mL/min. Before each catalytic test, the samples were pre-reduced at 850 °C for 1 h. The m/z signals of 16, 44 and 2 were analysed for determination of CH_4_, CO_2_ and H_2_ content in reaction mixture (upstream and downstream of the reactor).

Moreover, an m/z signal of 18 was used for analysis of water presence in the gas mixture. The content of CO was determined by analysis of m/z signal of 28. Because fragmentation of CO_2_ results in 8% of CO, therefore the CO content was determined by subtracting 8% of m/z = 44 from m/z = 28. The methane and carbon dioxide conversions were calculated based on the following formulas:

$$C_{CH_{4}}\left(T\right)=\frac{I_{CH_{4},in}-I_{CH_{4,out}}}{I_{CH_{4},in}}$$

$$C_{CO_{2}}\left(T\right)=\frac{I_{CO_{2},in}-I_{CO_{2,out}}}{I_{CO_{2},in}}$$

where

$I_{CH_{4},in}$—intensity of the m/z = 15 line for methane before the reactions occur

$I_{CH_{4},out}$—intensity of the m/z = 15 line for methane in the temperature T

$I_{CO_{2},in}$—intensity of the m/z = 44 line for carbon dioxide before the reactions occur

$I_{CO_{2},out}$—intensity of the m/z = 44 line for carbon dioxide in the temperature T

## 3. Results

**Figure 1:**
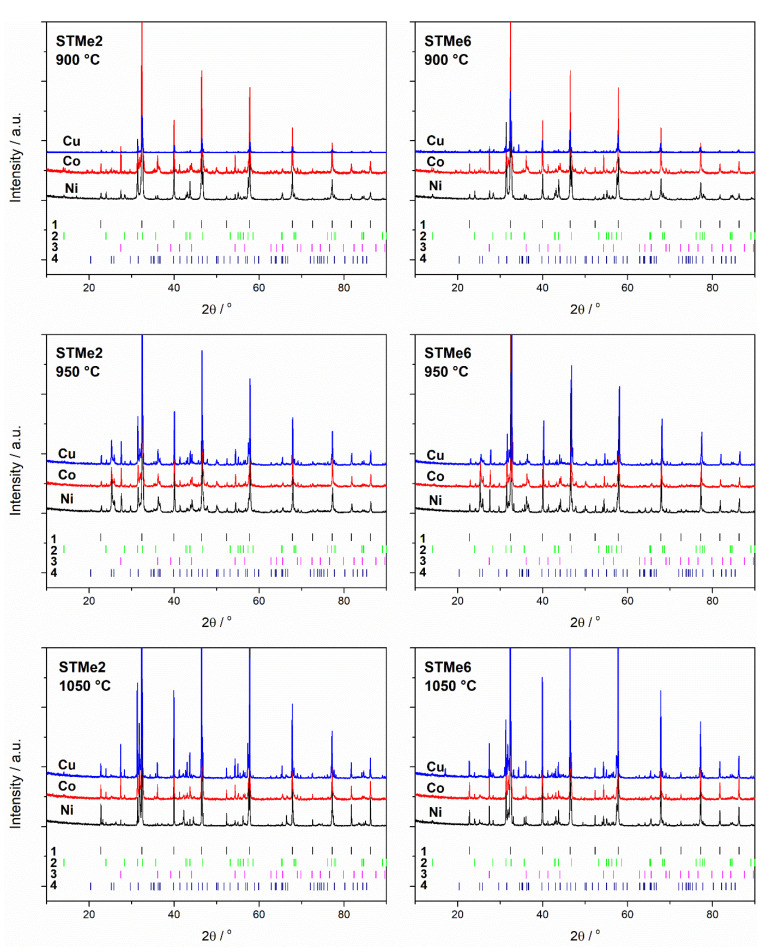
Diffraction patterns of STMe samples doped with 2 and 6 mol.% of Ni/Co/Cu calcined at different temperatures. Markers below the diffraction patterns numbered 1–4 represent tausonite (SrTiO_3_), Ruddlesden-Popper (RP) phase (Sr_2_TiO_4_), rutile (TiO_2_) and strontianite (SrCO_3_) phase respectively For clarity, the positions of reflections with a relative intensity of less than 2% have been removed from the patterns at the bottom of the figures.

The basic phase composition of both series of materials, STMe and SvTMe turned out to be similar, regardless of the calcination temperature and the amount of added admixture (Me). The main crystal phase identified in all the samples was tausonite (SrTiO_3_, ICCD no. 98-002-3076), the next identified in terms of quantity were R-P phases (Sr_2_TiO_4_, ICCD no. 98-015-7402 and in a lower quantity Sr_3_Ti_2_O_7_ ICCD no. 98-002-0294 or Sr_4_Ti_3_O_10_ ICDD no. 98-003-4630), while the phase present in smaller amount (up to 10 wt.%) was rutile (TiO_2_, ICCD no. 98-006-2679). The phases mentioned above were the only ones present in the case of materials with a lower content of Me admixture (2 mol.%), while for the materials with 6 mol.% of Me, the presence of additional phases was also identified. Diffractograms recorded for STMe materials, with 6 mol.% of cobalt/nickel/copper and calcinated at various temperatures were collected in Figure 1. In the case of STO doped with Ni and Cu, the presence of NiTiO_3_ (ICDD no. 98-004-4408) and Sr_2_CuO_3_/CuO (ICCD no. 98-015-1812/ no. 98-062-8614) phases was detected while for Co-doped STO no additional cobalt-containing phases were observed. Moreover, strontianite (SrCO_3_, ICCD no. 98-020-2793) in the case of the samples calcined at 950 °C was found, whereas for materials calcined at 900 °C and 1050 °C, its presence was not detected (Figure 1 and Table 1). The presence of SrCO_3_ only in powders calcinated at 950 °C is a surprising effect which, however, can be explained on the basis of studies on the SrO-CO_2_-SrCO_3_ system present in the literature [18,19]. According to the authors mentioned, the thermodynamics of SrO + CO_2_ ⇄ SrCO_3_ process depends greatly on the partial pressure of CO_2_. Moreover, with increase of temperature (in the temperature range 900–1175 °C) the equilibrium state shifts towards the carbonate decomposition. CO_2_ partial pressure can be determined not only by atmosphere pressure but also the pressure of CO_2_, which can be formed as a result of the oxidation of carbon deriving from organic residues. Thus, depending on the amount of carbon dioxide formed as a result of carbon oxidation, the equilibrium of the reaction shifts to the right or left. Considering the SrO-CO_2_-SrCO_3_ equilibrium diagram provided by Rhodes et al. it can be concluded that in the temperature range 900–1000 °C, the equilibrium is shifted towards the formation of strontium carbonate, while at higher temperatures, the preference for SrCO_3_ decomposition increases. It seems that the explanation for the lack of carbonate at 900 °C is that the CO_2_ pressure in the system at a low temperature is too low for carbonate formation.

The presence of Ni/Co/Cu oxides was not confirmed for a series with 2 mol.% of Me, nor for a series with 6% by XRD analysis. The only exception was the SvTMe6 sample, where the CuO phase was identified. However, on the basis of these observations, the presence of nickel, cobalt or copper oxides in the obtained materials cannot be excluded—their amounts may be below the detection limit of the XRD method. Another possible explanation is that the Me_x_O_y_ oxides can occur in amorphous form. The comparison of the phase composition of the STMe series and the SvTMe series does not indicate any general relationship resulting from strontium deficiency (non-stoichiometry) in relation to titanium or the metal introduced into its sublattice (Table 1).

However, it should be noted that the obtained materials were characterized by both small crystallite sizes, which causes the phase analysis error to be at the level of several %.

The crystallite sizes were determined from diffractometric measurements using the Scherrer method for tausonite as a major phase in the systems obtained (Table 2).

**Figure 2:**
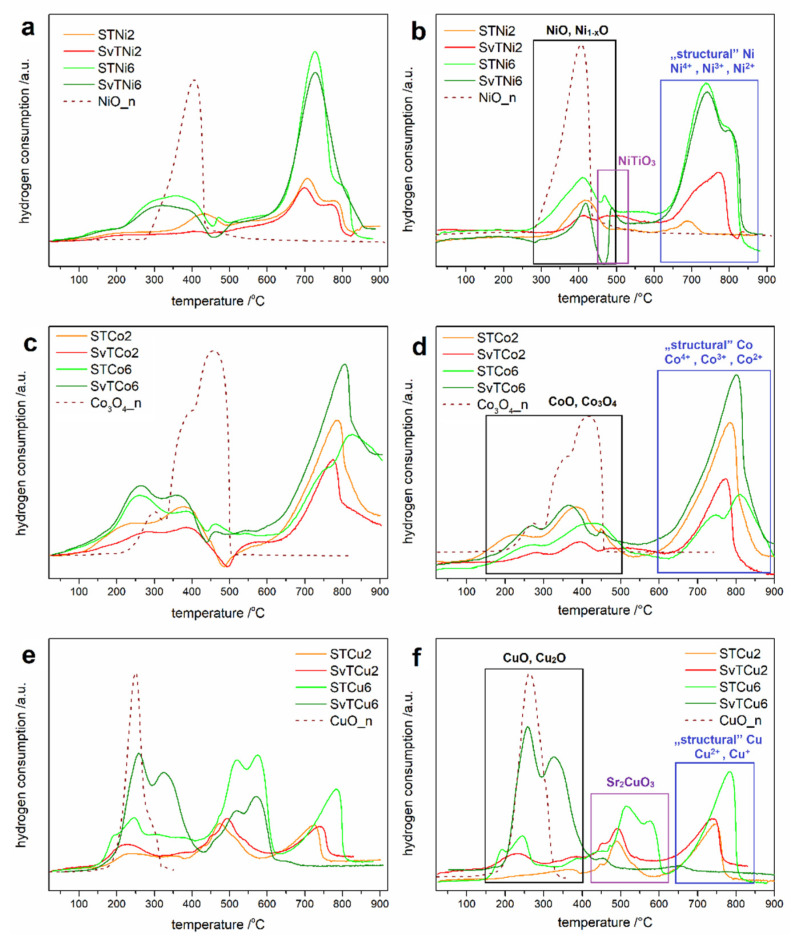
I TPR (**a**,**c**,**e**) and II TPR (**b**,**d**,**f**) profiles of STMe and SvTMe materials calcinated at 1050 °C.

Temperature-programmed reduction measurements provide information on the ability of a material to undergo redox reactions. For all tested materials, a series of measurements was carried out: reduction (I TPR), oxidation (TPOx), and reduction (II TPR). The results of the I and the II TPR cycles of the STMe and SvTMe materials are shown in Figure 2. The TPR profiles from the second TPR cycle are similar to the profiles registered for the first TPR cycle, i.e., the number of reduction peaks and their temperature range are consistent between the I and II TPR cycle. Only slight changes in the shape of the TPR profile may indicate a high redox stability of the tested materials. In our work, we only subjected the profiles of the second reduction to detailed analysis, treating the first reduction as a measurement that standardizes the system. The figures also show the reduction profiles (II TPR) of appropriate metal oxides (NiO, Co_3_O_4_ or CuO) as the reference materials. One can observe two temperature ranges of the reduction reactions on TPR profiles. The first one, in the low-temperature range, 250–450 °C, 150–450 °C and 150–400 °C for the reduction of NiO, Co_3_O_4_/CoO and CuO, respectively. These oxides are most likely in the form of grains or amorphous forms on the surface of the grains of tausonite and other phases. The second range at a higher temperature corresponds to the reduction of Ni, Co and Cu incorporated into tausonite crystal lattice, referred to in Figure 2 as “structural”. The research conducted by our team [20,21] on strontium titanate doping with nickel confirmed the presence of nickel in the titanium sublattice into SrTiO_3_ structure, and their results allow assigning high-temperature effects (on the II TPR profiles) to the reduction of nickel incorporated into perovskite structure. Therefore, by analogy to the previously conducted research on a system with nickel introduced into the SrTiO_3_ structure, high-temperature effects in the TPR profiles for STCo and STCu were assigned to the reduction processes of Co/Cu elements embedded into the structure of this perovskite. Since the incorporation of a part of Me into the structure of tausonite-derived phases (R-P phases) cannot be excluded, the high-temperature effects may also be related to the reduction of Me built into these phases.

Furthermore, between two low- and high-temperature ranges, for the Ni-doped and Cu-doped samples (Figure 2b,f), additional peaks can be observed on the TPR profiles. These peaks can be attributed to the reduction of NiTiO_3_ and Sr_2_CuO_3_, the presence of which was confirmed by XRD measurements. The presence of this region is not observed in the case of STCo/SvTCo materials, which is in line with the results of X-ray diffraction. According to XRD analysis of STCo/SvTCo materials, the cobalt-containing phases other than tausonite and R-P were observed.

**Figure 3:**
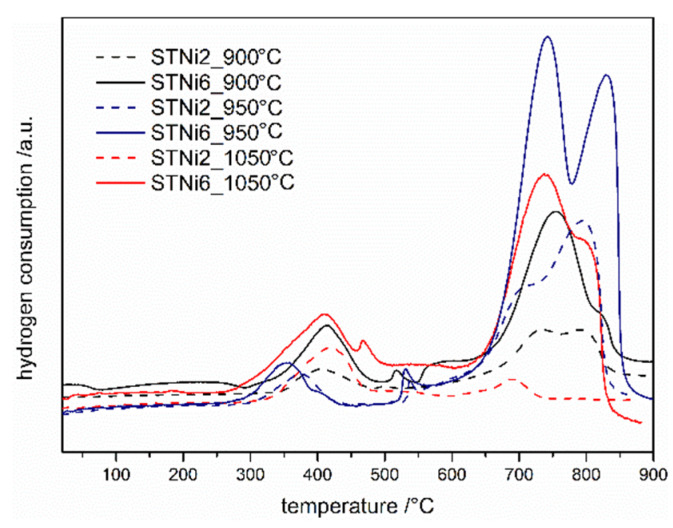
II TPR profiles of STNi materials calcinated at various temperature.

According to the literature data [22], the Ni-doped STO system should have the highest catalytic activity among the tested samples. This material’s ability to undergo reduction reactions was additionally tested in this system with different calcination temperatures (Figure 3). One can see that for all STNi materials, regardless of the calcination temperature, TPR profiles have similar shapes and reduction peak positions. Moreover, the peaks in the high temperature range have a greater intensity (and area) than those in the low temperature range for most of the samples. As could be expected, the total area of TPR peaks corresponding to the reduction of materials with a lower amount of nickel introduced (2%) is smaller than in the case of materials with 6% of nickel. Interestingly, the largest total TPR peak areas can be observed for TPR profiles corresponding to materials calcined at 950 °C. Taking into account the phase composition of the materials after calcination at 950 °C, it can be assumed that this effect is related to the presence of strontium carbonate in the materials. It can be suspected that during TPR/TPOx cycles, a partial thermal decomposition of SrCO_3_ occurs, which causes changes in the microstructure of the entire material. Thus, this results in an increase in the active (reactive) surface of various nickel-containing compounds, which are reduced. Thermal decomposition of SrCO_3_ can occur even below 900 °C [23], in the temperature range of temperature-programmed reduction/oxidation measurements.

TPR /TPOx studies indicate the high potential of the obtained STMe/SvTMe systems to participate in oxidation-reduction reactions, while the possibility of their real use in a specific reaction as catalysts must be verified by specific catalytic studies. As already mentioned, strontium titanate base-materials are systems with a high application potential in fuel cell technology. This potential is mainly from the electrical properties of these materials; however, the catalytic aspect of doped-SrTiO_3_ in fuel combustion reaction has not yet been specifically considered. Therefore, the manuscript presents the results of the SrTiO_3_-based systems used as catalysts for the DRM reaction, which is one of the basic reactions resulting in pure hydrogen that can be used as a fuel in the anode reaction in fuel cells. From application point of view, in the SOFC technology, the anodic material has to be sintered on the surface of solid electrolyte. In the majority of cases, sintering is a process carried out at temperatures above 1000 °C, necessary to permanently join the material grains. Thus, the samples were calcined not only below 1000 °C but also at 1050 °C to simulate the heat treatment process of manufacturing fuel cell anode-electrolyte composite material. As the catalysts for reforming reaction are metals (not oxides), before the tests in the DMR reaction, all tested STMe and SvTMe systems were reduced in a flow of hydrogen at 850 °C for an hour.

**Figure 4:**
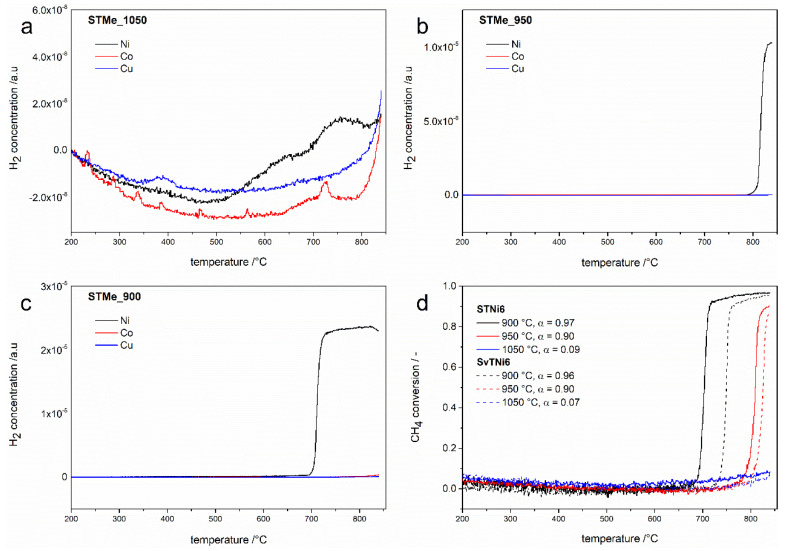
Concentration of hydrogen produced during catalytic tests for STMe6 samples calcined at different temperatures (**a**–**c**) and calculated conversion of methane for STNi6 and SvTNi6 materials calcined at different temperatures (**d**).

Catalytic tests were carried out for materials calcined at 900, 950 and 1050 °C (Figure 4). Since the main desired product of the DMR reaction is hydrogen, the results of the tests are presented in relation to this product.

As can be seen in Figure 4, among all the tested materials, only those doped with nickel (STNi and SvTNi) presented a catalytic activity in the dry reforming reaction. These results are consistent with the previously presented results of the DRM reaction [22,24], which reported nickel as the most effective component of active DRM catalysts (among non-noble metals). Copper and cobalt were less active in this reaction [24].

The effect of nickel activity in the DRM reaction can be observed for all STNi6 and SvTNi6 materials (after calcination at different temperatures) but with different intensities (Figure 4). The analysis of the hydrogen (formed as product of DRM) concentration profiles leads to the conclusion that the increase in calcination temperature of STNi and SvTNi materials results in decrease of the catalytic activity. This effect is the most evident for the results presented in Figure 4d, where the changes in the methane conversion are presented for the reaction carried out over the series of STNi and SvTNi materials calcined at different temperatures. The decrease in the catalytic activity of STNi and SvTNi materials calcined at a higher temperature is certainly related to the number of surface active sites, possibly nickel atoms, available for the reactants from gas phase. Their higher their number, the smaller the crystallites of the catalytically active phases (therefore the greater the specific surface area of the materials). The results of the crystallite sizes estimation (Table 2) clearly show that the materials calcined at 900 °C and 950 °C do not differ significantly, while an increase in calcination temperature to 1050 °C, increased these values twice in comparison to the samples calcined at lower temperatures. This is reflected in the results of the catalytic tests (Figure 4), which show that STNi6material calcined at 1050 °C presented a slight catalytic activity in the DMR reaction (the methane conversion degree is about 9%), while for the materials calcined at 900 °C and 950 °C, the degree of conversion reached 97% and 90%, respectively. Moreover, it is worth noting that the catalytic activity of materials with Sr-non-stoichiometry is similar to the catalytic activity of Sr-stoichiometric samples. A comparison of the CH_4_ and CO_2_ conversions for the Ni-doped strontium titanates with Ni catalysts from the literature data (with different support materials) is presented in Table 3.

The results presented above (the size of the crystallites and results of the catalytic tests) prove that the calcination temperature of the studied materials is a key parameter influencing their catalytic performance. An increase in the calcination temperature reduces the specific surface area of the catalyst and thus reduces the availability of catalytic centers on the catalyst surface. The agglomeration of nickel (as a reduction product of either oxide or other precursors) into larger particles under the influence of temperature is a known phenomenon. Moreover, the crystallites’ growth leads to a decrease in the amount of grain boundaries of material, which causes a decrease in system defectivity. The lowering of system defectivity can results in the reduction of Ni mobility into the perovskite structure. The above processes, together with an increase in the size of the crystallites, lead to a decrease in the reducibility of the catalyst and thus the catalytic activity.

The average values of specific surface areas (obtain on the basis of the BET method) of the STNi6 samples calcined at 900, 950 and 1050 °C were: 8.880 ± 0.500, 4.276 ± 0.021 and 3.150 ± 0.018 m^2^/g, respectively. The STCo and STCu samples derived as from lower as higher calcination temperatures showed no appreciable catalytic activity in the DRM reaction. This means that regardless of their specific surface, these materials are not effective catalysts in the DRM reaction. In our work, the yield of produced hydrogen is not given, but the axes have normalized units and therefore it is possible to compare these diagrams with each other.

**Figure 5:**
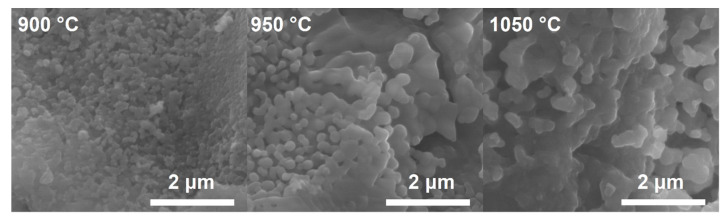
SEM micrographs of STNi6 samples from calcination at different temperatures (magnification 50,000×).

Selected samples were also observed under a scanning electron microscope (SEM). Micrographs are presented in Figure 5. The expected effect of increasing the calcination temperature of the materials is an increase in grain size, which can be seen in the SEM images. This confirms the results of the BET studies and the mean crystallite size estimated using the Scherrer formula obtained from the diffraction data.

**Figure 6:**
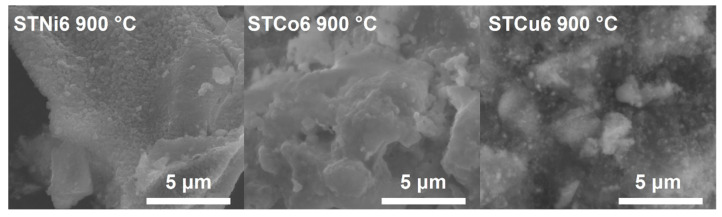
SEM micrographs of STMe6 samples from calcination at 900 °C (magnification 20,000×).

The comparison of the SEM micrographs of STMe6 materials calcined at 900 °C is presented in Figure 6. The microstructure of different STMe6 materials is similar; the materials are composed of the grains agglomerates.

**Figure 7:**
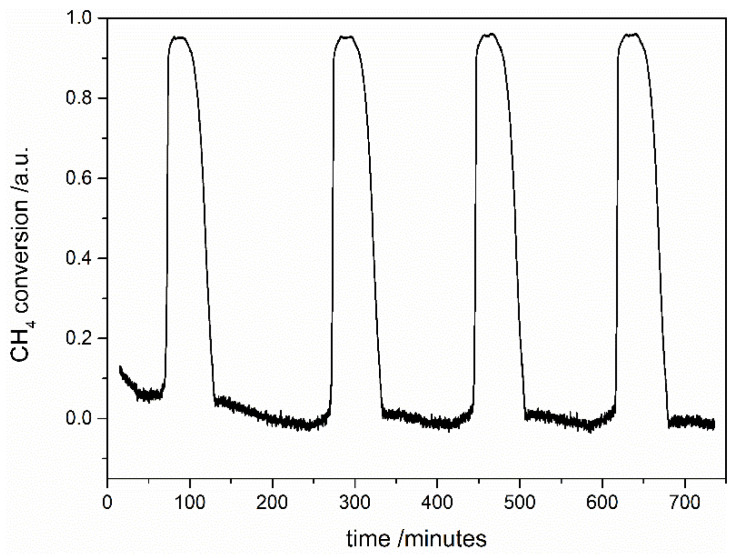
The conversion degree of methane on the STNi6 sample calcined at 900 °C during four cycles of the DRM reaction during heating and cooling of the sample.

Stability tests were performed for one chosen sample with the highest registered conversion degree of methane—the STNi6 sample calcined at 900 °C. The stability measurements were performed in the following way:

- The first stage was identical to the catalytic measurements in our studies: the sample was reduced and the catalytic test was performed during heating of the sample. After the test, the sample was cooled down in the He atmosphere;
- In the second step, the catalytic tests were performed during four cycles of heating up to 850 °C and cooling to the 200 °C of the sample. The conversion degree of methane from this step is presented in Figure 7;
- In the third stage, the catalytic test was performed, analogously to the first step.

We observed no significant difference between the first and the third stage of the stability tests. During heating and cooling of the samples (the second stage), the sample is stable and exhibits a comparable conversion degree of methane. Based on these results, we can conclude that the catalytic activity of STNi6 after six cycles of the DRM reaction does not deteriorate.

## 4. Conclusions

Two series of materials based on Ni/Co/Cu doped strontium titanate were obtained using the modified sol-gel method. The series differed in the strontium sublattice stoichiometry. Based on XRD measurements, the presence of tausonite (SrTiO_3_), Ruddlesden-Popper phases (Sr_2_TiO_4_, Sr_3_Ti_2_O_7_, Sr_4_Ti_3_O_10_), and rutile (TiO_2_) were found in almost all the samples except of SvTCu6 sample calcined at 1050 °C. Moreover, the crystalline phases containing admixture elements were detected only in the case of Ni or Cu-doped materials with a higher dopant content (NiTiO_3_ and Sr_2_CuO_3_, respectively). For a series calcined at 950 °C, strontianite (SrCO_3_) was also found. No correlation was found between the non-stoichiometry or the share of the admixture and the phase composition of the materials. XRD did not prove the presence of Ni, Co, Cu oxides, however, TPR/TPOx studies indicated their presence (the reduction of materials in the low-temperature range of 150–530 °C). Based on the II TPR profiles and our previous studies, the reduction of nickel, cobalt, and copper occurred at temperatures above 600 °C. The presence of the NiTiO_3_ and Sr_2_CuO_3_ phases is confirmed by the reduction peaks in the II TPR profile. The highest catalytic activity in the DRM reaction was observed for Ni-doped materials, which is in agreement with literature reports. Perovskites doped with Co and Cu presented the only slight effect of the CH_4_ conversion and thus were only poor active in the studied process. However, in the case of the Ni-doped catalysts, the catalytic activity decreased with increasing calcination temperature of the catalyst’s precursors. This effect can be explained by the growth of the grains and therefore a decrease in the surface area.

## Figures and Tables

**Table 1:** Phase composition of materials calcinated at 950 °C and 1050 °C in wt.%.

	Nickel		Cobalt		Copper	
	950 °C	1050 °C	950 °C	1050 °C	950 °C	1050 °C
STMe2	56.4% SrTiO_3_ 9.1% TiO_2_(r) 14.7%R-P 19.8% SrCO_3_	64.9% SrTiO_3_ 1.5% TiO_2_(r) 33.7% R-P	54.5% SrTiO_3_ 9.8% TiO_2_(r) 17.7% R-P 17.9% SrCO_3_	61.5% SrTiO_3_ 7.5% TiO_2_(r) 31.1% R-P	51.7% SrTiO_3_ 10.0% TiO_2_(r) 21.3% R-P 17.1% SrCO_3_	55.6% SrTiO_3_ 10.7% TiO_2_(r) 33.3% R-P 0.5% Sr_2_CuO_3_
STMe6	55.2% SrTiO_3_ 9.8% TiO_2_(r) 14.3% R-P 20.6% SrCO_3_	66.9% SrTiO_3_ 6.4% TiO_2_ (r) 23.7% R-P 2.9% NiTiO_3_	48.5%SrTiO_3_ 9.4% TiO_2_ (r) 22.6% R-P 19.5% SrCO_3_	66.7% SrTiO_3_ 5.9% TiO_2_(r) 27.5% R-P	57.9% SrTiO_3_ 8.7% TiO_2_(r) 20.2% R-P 13.2% SrCO_3_	64.2% SrTiO_3_ 9.2% TiO_2_(r) 25.9% R-P 2.6% Sr_2_CuO_3_
SvTMe2	46.3% SrTiO_3_ 10.2% TiO_2_ (r) 17.7% R-P 25.8% SrCO_3_	54.9% SrTiO_3_ 6.5% TiO_2_ (r) 39.6% R-P	67.6%SrTiO_3_ 9.2% TiO_2_ (r) 15.5% R-P 7.7% SrCO_3_	74.7% SrTiO_3_ 8.6% TiO_2_(r) 14.7% R-P	90.6% SrTiO_3_ 0.5% TiO_2_(r) 6.0% R-P 2.9% SrCO_3_	85.5% SrTiO_3_ 5.9% TiO_2_(r) 8.7% R-P
SvTMe6	54% SrTiO_3_ 11.3% TiO_2_ (r) 15.5% R-P 19.2% SrCO_3_	66.2% SrTiO_3_ 8.5% TiO_2_ (r) 22.1% R-P 3.1% NiTiO_3_	59.9%SrTiO_3_ 8.7% TiO_2_ (r) 18.8% R-P 12.6% SrCO_3_	68.1% SrTiO_3_ 8.1% TiO_2_(r) 23.8% R-P	73.2% SrTiO_3_ 7.4% TiO_2_(r) 9.7% R-P 9.6% SrCO_3_	97.9% SrTiO_3_2.1% CuO

**Table 2:** The values of crystallite sizes of tausonite for STMe materials.

Calcination Temperature/°C	Crystallite Size /nm					
	STNi2	STNi6	STCo2	STCo6	STCu2	STCu6
900 °C	7.0 ± 0.4	10.4 ± 0.8	5.4 ± 0.5	7.1 ± 0.6	5.0 ± 0.6	5.6 ± 1.2
950 °C	8.9 ± 0.7	14.3 ± 1.1	9.8 ± 0.7	11.0 ± 0.9	14.3 ± 1.2	12.4 ± 1.0
1050 °C	43.0 ± 3.5	25.1 ± 2.0	32.7 ± 2.2	36.7 ± 3.0	27.4 ± 2.2	31.1 ± 2.5

**Table 3:** Comparison of methane and carbon dioxide conversions in our study and chosen conversions from the literature.

Our Study				
Calcination temperature °C	900 °C	950 °C	1050 °C	
CH_4_ conversion				
STNi6	0.967	0.902	0.089	
SvTNi6	0.956	0.898	0.073	
CO_2_ conversion				
STNi6	0.850	0.759	0.280	
SvTNi6	0.854	0.762	0.451	
Literature data				
Wt. % of Ni/support	Preparation/calcination temperature	CH_4_ conversion	CO_2_ conversion	reference
10 Ni/Al_2_O_3_	Impregnation/550 °C	0.771	0.715	[25]
10 Ni/SiO_2_	Impregnation/550 °C	0.600	0.720	[25]
Ni/Al_2_O_3_-CeO_2_	Impregnation/750 °C	0.800	0.890	[26]

## Data Availability

The data presented in this study are available on request from the corresponding author.
